# Trend Analysis and Outcome Prediction in Mechanically Ventilated Patients: A Nationwide Population-Based Study in Taiwan

**DOI:** 10.1371/journal.pone.0122618

**Published:** 2015-04-13

**Authors:** Ming-Jang Lee, Chao-Ju Chen, King-Teh Lee, Hon-Yi Shi

**Affiliations:** 1 Department of Internal Medicine, Madou Sin-Lau Hospital, Tainan, Taiwan; 2 Department of Respiratory therepy, Madou Sin-Lau Hospital, Tainan, Taiwan; 3 Division of Hepato-biliary-pancreatic Surgery, Department of Surgery, Kaohsiung Medical University Hospital, Kaohsiung, Taiwan; 4 Department of Healthcare Administration and Medical Informatics, Kaohsiung Medical University, Kaohsiung, Taiwan; Hospital Sirio-Libanes, BRAZIL

## Abstract

**Objective:**

To investigate the relationship between changes in patient attributes and hospital attributes over time and to explore predictors of medical utilization and mortality rates in mechanical ventilation (MV) patients in Taiwan.

**Background:**

Providing effective medical care for MV patients is challenging and requires good planning and effective clinical decision making policies. Most studies of MV, however, have only analyzed a single regional ventilator weaning center or respiratory care unit, high-quality population-based studies of MV trends and outcomes are scarce.

**Methods:**

This population-based cohort study retrospectively analyzed 213,945 MV patients treated during 2004-2009.

**Results:**

During the study period, the percentages of MV patients with the following characteristics significantly increased: age ≦ 65 years, treatment at a medical center, and treatment by a high-volume physician. In contrast, the percentages of MV patients treated at local hospitals and by low-volume physicians significantly decreased (*P*<0.001). Age, gender, Deyo-Charlson co-morbidity index, teaching hospital, hospital level, hospital volume, and physician volume were significantly associated with MV outcome (*P*<0.001). Over the 6-year period analyzed in this study, the estimated mean hospital treatment cost increased 48.8% whereas mean length of stay decreased 13.9%. The estimated mean overall survival time for MV patients was 16.4 months (SD 0.4 months), and the overall in-hospital 1-, 3-, and 5-year survival rates were 61.0%, 36.7%, 17.3%, and 9.6%, respectively.

**Conclusions:**

These population-based data revealed increases in the percentages of MV patients treated at medical centers and by high-volume physicians, especially in younger patients. Notably, although LOS for MV patients decreased, hospital treatment costs increased. Healthcare providers and patients should recognize that attributes of both the patient and the hospital may affect outcomes.

## Introduction

Mechanical ventilation (MV) is among the most common therapeutic interventions in intensive care units (ICUs), and the use of MV tends to be highest in developed countries [1]. As economic conditions increase pressure to improve efficiency in medical resource utilization, hospitals have begun transferring patients who require MV but who are hemodynamically stable from ICUs into other hospital wards or other facilities [2]. A retrospective cohort study of 15,757 adult patients in 361 ICUs in the United States and other Organisation for Economic Co-operation and Development (OECD) countries over a 31-day period revealed that 33% had received MV for a mean duration of 5.9 days [3]. Mechanical ventilation can cause substantial and often lifelong cognitive, physical and behavioral impairments that require long-term access to healthcare services [4–6]. However, predicting the healthcare service utilization and mortality rate associated with MV is difficult because of its widely varying rate and extent of recovery. Additionally, although MV patients consume substantial medical resources, their outcomes tend to be poor, especially in the rapidly growing elderly population.

Most studies of MV, however, have only analyzed a single regional ventilator weaning center or respiratory care unit, which may not provide an objective estimation. Furthermore, although many studies have evaluated MV outcomes, few longitudinal studies have exceeded 10 years, and most published data have been limited to populations in the United States or in OECD countries. Additionally, very few studies of MV patients have performed longitudinal analyses of survival and temporal trends in hospital resource utilization, and none have systematically evaluated associations with hospital resource utilization and survival in this patient group.

Providing effective medical care for MV patients is challenging and requires good planning and effective clinical decision making policies. Thus, the aim of this population-based cohort study was to investigate the relationship between changes in patient attributes and hospital attributes over time and to explore predictors of hospital resource utilization and mortality rates in a nationwide MV population.

## Materials and Methods

### Ethics statement

This study analyzed administrative claims data obtained from the Taiwan Bureau of National Health Insurance (BNHI). The Institutional Review Board of the Kaohsiung Medical University Hospital approved this study in Taiwan. Written consent from study patients was not obtained because the NHI dataset consists of de-identified secondary data for research purposes, and the Institutional Review Board of Kaohsiung Medical University Hospital issued a formal written waiver for the need for consent. Additionally, because the BNHI is the sole payer in Taiwan, the BNHI data set was assumedly the most comprehensive and reliable data source for the study. The subjects of this study were recruited by reviewing monthly patient discharge data released by the BNHI. The database contains a registry of contracted medical facilities, a registry of board-certified physicians, and monthly summaries for all inpatient claims.

### Study patients

The analysis included records for patients who had received an International Classification of Diseases, 9th Revision, Clinical Modification (ICD-9-CM) diagnosis code for MV (96.70, period of ventilation unspecified; 96.71, ventilated for less than 96 hours; 96.72, ventilated for longer than 96 hours). The assessment was limited to patients who had received ventilation for longer than 96 hours [7]. The purposes of this criterion were to limit the analysis to patients with treatment times sufficiently long to evaluate overall patient care management techniques and practice patterns and to increase the homogeneity of the studied patient population among different facilities and over time [8]. After excluding data for patients aged younger than 18 years and patients with continuous MV shorter than 96 consecutive hours (n = 417,400 subjects), the final dataset included data for 213,945 MV patients treated from 2004 to 2009.

### Potential confounders

The attributes analyzed in this Taiwan population of MV patients were age, gender, co-morbidity, teaching hospital, hospital type, hospital level, hospital volume, physician volume, length of stay (LOS), and hospital treatment cost. The analysis also included any co-morbidity such as infectious and parasitic diseases; tumors; endocrine, nutritional, or metabolic diseases or immunity disorders; mental system and sensory organ diseases; circulatory system diseases; digestive system diseases; genitourinary system diseases; skin and subcutaneous tissue disorders; symptoms: signs and unexplained; and injury and poisoning. Age categories were ≦ 65 years and > 65 years. Co-morbidities were identified by ICD-9-CM codes, which were used to calculate the Deyo-Charlson co-morbidity index (CCI) [9]. In accordance with the criteria used by the Taiwan Joint Commission on Hospital Accreditation, hospital level was classified as medical center (> 500 beds), regional hospital (> 300 beds), or local hospital (> 100 beds). Hospital volume or physician volume was categorized as low and high if the number of patients who had received MV by the hospital or by the physician in the previous year was ≦50% and > 50%, respectively, of the total patients treated by the hospital or by the physician that year.

### Statistical analyses

The unit of analysis in this study was the individual MV patient. The characteristics of the study patients were expressed in terms of sample size (percentage) or median (interquartile range, IQR). Trends in the prevalence of MV patients were analyzed by Cochrane-Armitage trend test. The study period was divided into three equal time intervals (period 1: 2004~2005; period 2: 2006~2007; and period 3: 2008~2009). Odds ratio (OR) and 95% confidence interval (95% C.I.) were determined to assess the temporal trend in each factor when using period 1 as the reference group in comparison with period 3. Hospital treatment costs were analyzed by collecting data for the following medical costs, which are required data in standard administrative claims for reimbursement from the Taiwan BNHI: operating room, radiology, physical therapy, hospital room, anesthetist, pharmacy, laboratory, special materials, surgeon, and others. Hospital treatment cost was adjusted for specific hospital levels according to their differences in BNHI reimbursements. To reflect changes in real dollar value, cost data were also adjusted by the consumer price index (CPI) for each year of 2004–2009. Hospital treatment costs were then converted from Taiwan dollars to US dollars at an exchange rate of 30.5:1, which was the average exchange rate during 2004–2009.

In comparisons of different groups with the reference group in terms of the relationship between LOS and hospital treatment cost, hierarchical linear regression method was used to exclude the potential hospital clustering effect (i.e., to exclude the effects of policies, procedures, or surgeon compensation mechanisms unique to each hospital on the cost and quality of health care) [9]. All potential covariates were assessed for collinearity, defined as a variance inflation factor (VIF) >10, and the more relevant of these variables was included, whereas the collinear variable was excluded in the final regression model. Overall survival time was calculated from the date of the MV to the date of the last recorded hospital admission. Kaplan–Meier survival curves to estimate overall survival were generated and compared by using the log rank test. Survival is expressed as median [IQR]. The Cox proportional hazard models were employed to identify predictors of both 90-day mortality and 5-year mortality within the cohort. The proportional hazard assumption was tested by plotting scaled Schoenfeld residuals against time and testing for a zero slope [10]. In the event of violation of proportional hazard assumption for a specific covariate, the same covariate was included as a stratifying factor in the Cox model. The correlations between effective predictors and mortality rates are expressed as hazard ratios (HRs) and 95% CIs.

Statistical analyses were performed using SPSS version 19.0 (SPSS Inc., Chicago, IL, USA). All tests were two-sided, and p values less than 0.05 were considered statistically significant.

## Results

**Fig 1:**
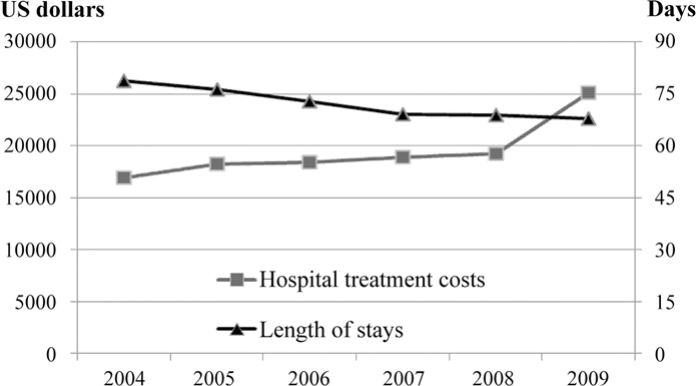
Trends in hospital treatment cost and length of stay for mechanically ventilated (MV) patients during the study period.

The prevalence rate of MV patients was 138.5 per 100,000 persons in 2004 and significantly increased to 182.5 per 100,000 persons by 2009 (data not shown). Thus, the rate of increase from 2004 to 2009 was 31.8%, which was statistically significant (*P*<0.001). The estimated mean hospital treatment cost significantly (*P*<0.001) increased from $16,884 in 2004 to $25,123 in 2009, which was a 48.8% increase (Fig 1). Conversely, the LOS decreased from 78.8 days in 2004 to 67.9 days in 2009, which was a 13.9% decrease.

Regarding the patient characteristics in this study, the mean age was 73.5 years (standard deviation (SD) 13.9 years), and 61.0% of the patients were male (Table 1). The mean CCI score was 1.5 (SD = 1.5 points). The estimated mean LOS was 97.0 days (SD 18.2 days), and the mean hospital treatment cost was $19,708 (SD $12,431). Table 2 shows the results of the trend analysis of study characteristics from periods 1 to 3. The percentage of MV patients aged ≦ 65 years old significantly increased from 19.9% to 20.8%. Moreover, during the same period, significant increases were noted in the percentages of patients who had infectious and parasitic diseases (33.9% to 37.3%), tumor (10.6% to 10.9%), symptoms: signs and unexplained (19.1% to 22.5%), and injury and poisoning (8.7% to 9.8%). Conversely, significant decreases were noted in the percentages of patients with circulatory system diseases (48.3% to 47.1%), digestive system diseases (22.0% to 19.6%), genitourinary system diseases (37.3% to 35.9%), and skin and subcutaneous tissue disorders (7.7% to 6.7%). Additionally, the percentage of patients treated at a teaching hospital significantly increased from 71.5% to 73.4%, and the percentage of patients treated at a medical center significantly increased from 26.0% to 27.2%. In contrast, the percentage of patients treated at a local hospital significantly decreased from 35.7% to 34.0%. The analysis of physician volume showed that the percentage of patients treated by high-volume physicians significantly increased from 48.8% to 49.5%, but the percentage of patients treated by low-volume physicians significantly decreased from 51.2% to 50.5%.

**Table 1:** Patient characteristics (N = 213,945).

Variables	Number	%
Age group (years)*	77 [68–83]	
≦65	43,376	20.3
>65	170,569	79.7
Gender		
Male	130,506	61.0
Female	83,439	39.0
Infectious and parasitic diseases		
Yes	76,975	36.0
Tumor		
Yes	23,407	10.9
Endocrine, nutritional, and metabolic diseases and immunity disorders		
Yes	63,653	29.8
Mental system and sensory organ diseases		
Yes	23,617	11.0
Circulatory system diseases		
Yes	101,788	47.6
Digestive system diseases		
Yes	44,601	20.8
Genitourinary system diseases		
Yes	78,213	36.6
Skin and subcutaneous tissue disorders		
Yes	15,479	7.2
Symptoms: signs and unexplained		
Yes	45,131	21.1
Injury and poisoning		
Yes	19,746	9.2
Teaching hospital		
Yes	155,708	72.8
Hospital level		
Medical center	58,352	27.3
Region hospital	81,322	38.0
Local hospital	74,271	34.7
Hospital volume (cases/year)*	219 [106–438]	
Low	107,930	50.5
High	106,015	49.5
Physician volume (cases/year)*	26 [8–56]	
Low	108,784	50.9
High	105,161	49.1
	Mean	Standard deviation
Mean age (years)	73.5	13.9
Charlson comorbidity index (score)*	1.5	1.5
Length of stay (days)	97.0	18.2
Hospital treatment cost (US dollars)	19,708.3	12,431.8

IQR, interquartile range.

*Median (IQR[range])

**Table 2:** Changing trends in patient demographics and hospital data.

Variables	T1 (N = 65,656)	T2 (N = 69,719)	T3 (N = 78,570)	OR (95% C.I.)*
Age group				
≦65	19.9%	20.1%	20.8%	1.05 (1.02, 1.08)
>65	80.1%	79.9%	79.2%	0.99 (0.97, 1.00)
Gender				
Male	60.8%	61.4%	60.8%	1.00 (0.98, 1.02)
Female	39.2%	38.6%	39.2%	1.00 (0.98, 1.02)
Infectious and parasitic diseases				
Yes	33.9%	36.5%	37.3%	1.10 (1.08, 1.12)
Tumor				
Yes	10.6%	11.1%	10.9%	1.06 (1.02, 1.09)
Endocrine, nutritional, and metabolic diseases and immunity disorders				
Yes	30.1%	29.3%	29.9%	0.99 (0.97, 1.02)
Mental system and sensory organ diseases				
Yes	11.0%	10.9%	11.2%	1.02 (0.99, 1.06)
Circulatory system diseases				
Yes	48.3%	47.4%	47.1%	0.98 (0.96, 0.99)
Digestive system diseases				
Yes	22.0%	21.1%	19.6%	0.89 (0.87, 0.92)
Genitourinary system diseases				
Yes	37.3%	36.6%	35.9%	0.96 (0.94, 0.98)
Skin and subcutaneous tissue disorders				
Yes	7.7%	7.4%	6.7%	0.87 (0.83, 0.90)
Symptoms: signs and unexplained				
Yes	19.1%	21.4%	22.5%	1.18 (1.15, 1.21)
Injury and poisoning				
Yes	8.7%	9.1%	9.8%	1.12 (1.08, 1.16)
Teaching hospital				
Yes	71.5%	73.4%	73.4%	1.03 (1.01, 1.04)
Hospital level				
Medical center	26.0%	28.6%	27.2%	1.04 (1.02, 1.07)
Region hospital	38.3%	36.8%	38.8%	1.01 (0.99, 1.03)
Local hospital	35.7%	34.6%	34.0%	0.95 (0.93, 0.97)
Hospital volume (cases/year)*				
Low	50.3%	50.7%	50.3%	0.99 (0.97, 1.01)
High	49.7%	49.3%	49.7%	0.99 (0.97, 1.01)
Physician volume (cases/year)*				
Low	51.2%	51.1%	50.5%	0.98 (0.97, 0.99)
High	48.8%	48.9%	49.5%	1.02 (1.01, 1.04)

*T3 (2006–2009) versus T1 (1998–2001) (reference group)

The hierarchical linear regression model showed the largest significant decreases in mean LOS and mean hospital treatment cost in high-volume hospitals compared to low-volume hospitals (decreases of 60.03 days and $6,118, respectively; *P*<0.001) (Table 3). Average LOS and hospital treatment cost for high-volume hospitals was 57.9% shorter and 21.6% lower, respectively, compared to low-volume hospitals. Moreover, mean LOS and hospital treatment cost in high-volume physicians was significantly shorter than that in low-volume physicians (45.8% shorter and 17.3% lower, respectively; *P*<0.001). Long LOS concurrent with a high hospital treatment cost also revealed significant (*P<*0.05) associations with the following attributes: advanced age, male gender, high CCI score, treatment at a non-teaching hospital, treatment at a regional hospital or a local hospital, treatment at a low-volume hospital, treatment by a low-volume physician, and any of the following co-morbidities: infectious and parasitic diseases, tumor, endocrine, nutritional, or metabolic diseases or immunity disorders, mental system and sensory organ diseases, circulatory system diseases, digestive system diseases, genitourinary system diseases, skin and subcutaneous tissue disorders, symptoms: signs and unexplained, and injury and poisoning.

**Table 3:** Hierarchical linear regression model for effective predictors of length of stay and hospital treatment cost.

	Length of stay			Hospital treatment cost		
Variables	Coefficients	Standard error	P value	Coefficients	Standard error	P value
Age group (years)						
>65 vs. ≦65	8.62	0.99	<0.001	1,712.61	133.57	<0.001
Gender								
Male vs. female	11.82	0.81	<0.001	1,513.01	108.36	<0.001
Charlson comorbidity index	1.15	0.33	0.001	207.94	44.96	<0.001
Teaching hospital						
Yes vs. no	-58.44	1.58	<0.001	-4,950.74	212.59	<0.001
Hospital level						
Regional hospital vs. medical center	4.55	1.33	0.001	2,466.68	179.15	<0.001
Local hospital vs. medical center	14.76	2.07	<0.001	3,559.12	279.03	<0.001
Hospital volume						
High vs. low	-60.03	2.50	<0.001	-6,118.24	302.20	<0.001
Physician volume						
High vs. low	-48.29	2.13	<0.001	-5,038.40	252.52	<0.001
Infectious and parasitic diseases						
Yes vs. no	5.07	0.88	<0.001	893.39	118.97	<0.001
Tumor						
Yes vs. no	11.57	1.56	<0.001	524.87	210.35	0.013
Endocrine, nutritional, and metabolic diseases and immunity disorders						
Yes vs. no	1.95	0.90	0.031	181.82	121.51	0.135
Mental system and sensory organ diseases						
Yes vs. no	27.76	1.28	<0.001	3,221.476	172.50	<0.001
Circulatory system diseases						
Yes vs. no	10.62	0.89	<0.001	1,376.70	119.43	<0.001
Digestive system diseases						
Yes vs. no	2.35	1.06	0.026	654.16	141.95	<0.001
Genitourinary system diseases						
Yes vs. no	1.12	0.84	0.185	565.81	113.27	<0.001
Skin and subcutaneous tissue disorders						
Yes vs. no	6.97	1.52	<0.001	1,154.94	204.53	<0.001
Symptoms: signs and unexplained						
Yes vs. no	0.54	1.01	0.595	687.20	135.40	<0.001
Injury and poisoning						
Yes vs. no	7.39	1.41	<0.001	2,882.79	190.28	<0.001
Residual variance	67.63			24,548,126.57		
Random effect associated with hospital	4.02			468,318.91		
Constant	21.27	0.42	<0.001	3,684.29	132.78	<0.001

**Fig 2:**
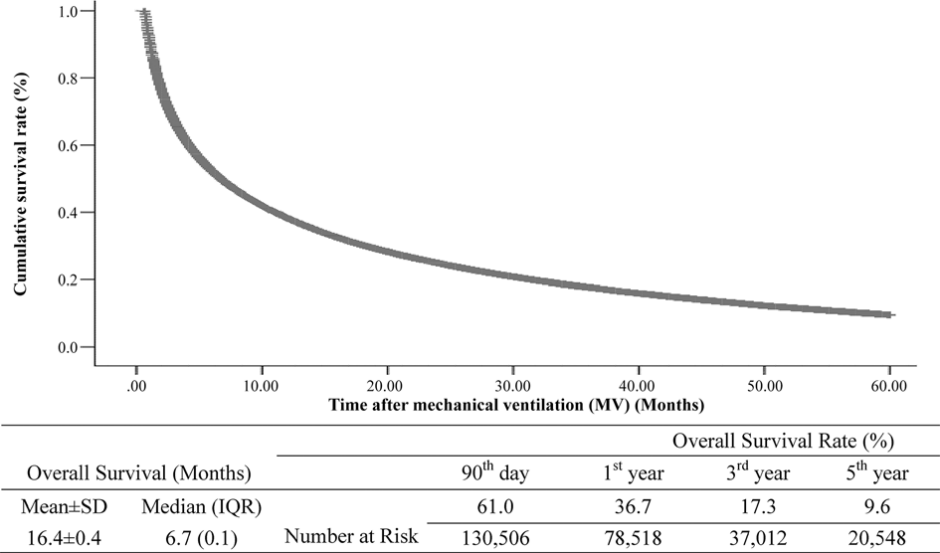
Cumulative overall survival rate for mechanically ventilated (MV) patients during the study period.

Multivariate Cox regression analysis showed that 90-day mortality and 5-year mortality were significantly (*P*<0.05) associated with advanced age, male gender, high CCI score, treatment at a regional hospital or a local hospital, treatment at a low-volume hospital, treatment by a low-volume physician, and any of the following co-morbidities: infectious and parasitic diseases, tumor, endocrine, nutritional, or metabolic diseases or immunity disorders, mental system and sensory organ diseases, circulatory system diseases, digestive system diseases, genitourinary system diseases, skin and subcutaneous tissue disorders, symptoms: signs and unexplained, and injury and poisoning (Table 4). Additionally, after adjusting all covariates, the estimated mean and median overall survival times for MV patients were 16.4 months (SD 0.4 months) and 6.7 months (IQR 0.1 months), respectively (Fig 2). The overall survival rates at 90 days, 1 year, 3 years, and 5 years were 61.0%, 36.7%, 17.3%, and 9.6%, respectively.

**Table 4:** Cox regression model of relationships of effective predictors to 90-day mortality and 5-year mortality.

	90-day mortality		5-year mortality	
Variables	HR	P value	HR	P value
Age group (years)				
>65 vs. ≦65	1.29	<0.001	1.65	<0.001
Gender				
Male vs. female	1.09	<0.001	1.13	<0.001
Charlson comorbidity index	1.10	<0.001	1.09	<0.001
Teaching hospital				
Yes vs. no	0.93	0.411	0.92	0.340
Hospital level				
Region hospital vs. medical center	1.03	0.028	1.06	<0.001
Local hospital vs. medical center	1.00	0.827	1.09	<0.001
Hospital volume				
High vs. low	0.84	<0.001	0.89	<0.001
Physician volume				
High vs. low	0.87	<0.001	0.91	0.001
Infectious and parasitic diseases				
Yes vs. no	1.60	<0.001	1.35	<0.001
Tumor				
Yes vs. no	1.85	<0.001	1.73	<0.001
Endocrine, nutritional, and metabolic diseases and immunity disorders				
Yes vs. no	1.49	<0.001	1.09	<0.001
Mental system and sensory organ diseases				
Yes vs. no	1.70	<0.001	1.78	<0.001
Circulatory system diseases				
Yes vs. no	1.09	<0.001	1.31	<0.001
Digestive system diseases				
Yes vs. no	1.08	<0.001	1.15	<0.001
Genitourinary system diseases				
Yes vs. no	1.11	<0.001	1.12	<0.001
Skin and subcutaneous tissue disorders				
Yes vs. no	1.09	0.004	1.10	<0.001
Symptoms: signs and unexplained				
Yes vs. no	1.48	<0.001	1.27	<0.001
Injury and poisoning				
Yes vs. no	1.84	<0.001	1.82	<0.001

## Discussion

This study is the first to use a nationwide population-based follow-up design to analyze long-term trends and predictors of hospital resource utilization and mortality in a population of MV patients. Annual increases in the prevalence of MV patients were observed during 2004–2009. The data also confirmed previous reports that MV treatment outcomes are associated with the following: age, gender, CCI score, teaching hospital, hospital level, hospital volume, physician volume, infectious and parasitic diseases, tumor, endocrine, nutritional, or metabolic diseases or immunity disorders, mental system and sensory organ diseases, circulatory system diseases, digestive system diseases, genitourinary system diseases, skin and subcutaneous tissue disorders, symptoms: signs and unexplained, and injury and poisoning [3, 7, 8, 11–13]. Our findings highlight the continuing need for efforts to increase the efficiency of medical resource utilization, to improve clinical outcomes in this population, and to provide renewed justification for prioritizing acute respiratory failure and MV in national research and policy initiatives.

The convenience and accessibility of the Taiwan national health insurance system may increase the likelihood of treatment for MV patients [1, 2]. Improved medical techniques may also increase the number of patients with co-morbidities who are willing to receive invasive MV [14]. The prevalence of MV patients younger than 65 years has also increased, and patients in this age group tend to prefer treatment in high-volume hospitals or medical centers [1, 2].

This analysis of trends in MV patients showed that, during 2004–2009, hospital treatment cost increased, but LOS decreased. These higher hospital treatment costs were apparently driven mainly by the complex interplay between case mix and the use of high medical technology. These results mirror the findings of other studies indicating that the incidence of critical illness syndromes such as sepsis and acute lung injury increases older populations [15]. Indeed, most patients in the present study were aged 65 years or older. Advances in medical technology have also increased the selection of treatment options and have improved medical techniques and instruments, management of major treatments, and the quality hospital medical care, all of which have achieved decreased morbidity rates, mortality rates, and LOS in MV patients. Notably, however, although both of these trends have contributed to increased treatment options and increased availability of medical resources, they have also increased hospital treatment costs.

This study also found that LOS, hospitalization cost, 90-day mortality, and 5-year mortality were significantly lower in MV patients treated at high-volume hospitals and by high-volume physicians compared to those treated at low-volume hospitals and by low-volume physicians. Hospital treatment costs were 21.6% lower in high-volume hospitals than in low-volume hospitals and 17.3% lower in high-volume physicians than in low-volume physicians. Therefore, our data suggest that, when allocating limited emergency medical resources to MV, emergency transfer systems should consider not only the potential improvement in outcomes, but also the economies of scale achieved by high-volume hospitals and high-volume physicians.

As in previous reports, our study also showed that LOS was significantly lower in patients treated in high-volume hospitals than in those treated in low-volume hospitals. Furthermore, advanced age reportedly has a strong positive association with a high CCI score and medical resource utilization and mortality rate tend to be higher in MV patients with several co-morbidities [7, 16]. This may explain why the current study revealed long LOS, high hospital treatment cost and high mortality rates in MV patients aged 65 and over who had infectious and parasitic diseases, tumor, endocrine, nutritional, or metabolic diseases or immunity disorders, mental system and sensory organ diseases, circulatory system diseases, digestive system diseases, genitourinary system diseases, skin and subcutaneous tissue disorders, symptoms: signs and unexplained, or injury and poisoning. This may explain why the mean LOS of the current study was longer than that reported in comparable populations elsewhere and it deserves a further study. Additionally, this study also confirms the volume-outcome relationship in MV patients and the synergistic effects of hospital volume and physician volume. The low LOS, hospital treatment cost and short- or long-term mortality rate observed in MV patients treated by high-volume physicians in the previous year and the positive association observed between MV outcomes and volumes of treatments performed by the physician and by the hospital in the past year are consistent with other cross-sectional studies [1, 16, 17]. Medical outcomes clearly depend not only on patient management, but also on the skill and experience of individual physicians. Meanwhile, high-volume physicians in high-volume hospitals are most likely to achieve good patient outcomes because they are assisted by highly skilled and interdisciplinary medical teams [18].

In the current study, the trend analysis of MV revealed an increasing hospital treatment cost, a decreasing LOS, and an increasing temporal trend in the prevalence of MV, especially in younger patients. All three trends were simultaneous and consistent over time. Notably, the results are the opposite of those reported in America, where the incidence of MV patients discharged to long-term acute care is increasing [19, 20]. Apparently, the percentage of MV patients treated at high-volume hospitals during the same time period was higher in Taiwan than in the United States. The potential benefits of MV observed in the elderly patients analyzed in the current study may impact the management and treatment approaches applied in other countries where medical teams are adequately equipped and trained to perform MV treatment [1, 7]. Improved medical techniques and recent advances in medical technology may explain the progressively improving outcomes reported in the literature [1, 19, 20].

This study has several limitations that are inherent in any large database analysis. The population-based study design minimises ascertainment bias and allows for outcomes prediction analysis. Several investigators who examined the temporal trends and outcomes prediction of MV patients have previously confirmed their accuracy, validity, and reliability in the NHI database [1, 4]. Firstly, the clinical picture obtained by analyzing claims data is not as precise as that obtained by analyzing prospective clinical trial data due to possible errors in the coding of primary diagnoses and treatment modalities. The relationship between these factors and hospital resource utilization and mortality rate could not be evaluated. Secondly, complications associated with hospital resource utilization and mortality rate were not assessed, which limits the validity of the predictions. Thirdly, the main study limitation was the lack of acute physiology and chronic health evaluation (APACHE) score, which is an important factor on the outcomes. So, the correlation between APACHE II of the subjects and outcomes could not be established. However, the patients enrolled in this study were indicated for MV, they were selected from those with moderate to severe MV. The patients with low-risk conditions or those with very severe respiratory diseases and sepsis, were not considered for MV. Thus, the result of this study could demonstrate the effective predictors and outcomes of MV patients, despite the lack of APACHE II. Finally, the analysis did not examine outcome data such as patient-reported quality of life and indirect costs incurred after discharge. However, given the robust magnitude of the effects and the statistically significant effects observed in this study, these limitations are unlikely to compromise the results.

## Conclusions

In conclusion, this study showed that a population-based study of MV is feasible. Despite the huge and growing global burden of MV, high-quality population-based studies of MV prevalence and outcomes are scarce. The data analysis revealed an increased prevalence of MV, especially in younger patients. Moreover, hospital treatment cost increased, but LOS decreased. Healthcare providers and patients should also understand that hospital resource utilization and mortality rates depend not only on patient attributes, but also on hospital attributes. Additionally, similar population-based studies of MV incidence, causes, and outcomes in other populations are urgently needed to establish reliable surveillance systems for monitoring and evaluating intervention strategies, for implementing evidence-based health-care planning, and for developing effective treatment, prevention, and rehabilitation strategies.
